# IoT Elements, Layered Architectures and Security Issues: A Comprehensive Survey

**DOI:** 10.3390/s18092796

**Published:** 2018-08-24

**Authors:** Muhammad Burhan, Rana Asif Rehman, Bilal Khan, Byung-Seo Kim

**Affiliations:** 1 Department of Computer Science, National University of Computer and Emerging Sciences, Chiniot-Fasialabad Campus, Chiniot 35400, Pakistan; f169033@nu.edu.pk (M.B.); r.asif@nu.edu.pk (R.A.R.); khan.bilal@nu.edu.pk (B.K.); 2 Department of Software and Communications Engineering, Hongik University, Sejong City 30016, Korea

**Keywords:** Internet of Things (IoT), layered architectures, security, privacy, security attacks, protection methods, secure architecture

## Abstract

The use of the Internet is growing in this day and age, so another area has developed to use the Internet, called Internet of Things (IoT). It facilitates the machines and objects to communicate, compute and coordinate with each other. It is an enabler for the intelligence affixed to several essential features of the modern world, such as homes, hospitals, buildings, transports and cities. The security and privacy are some of the critical issues related to the wide application of IoT. Therefore, these issues prevent the wide adoption of the IoT. In this paper, we are presenting an overview about different layered architectures of IoT and attacks regarding security from the perspective of layers. In addition, a review of mechanisms that provide solutions to these issues is presented with their limitations. Furthermore, we have suggested a new secure layered architecture of IoT to overcome these issues.

## 1. Introduction

The Internet has become a basic need of millions of people who use it for many purposes according to their needs. People not only use the Internet for entertainment (movies, songs and games) but also to fulfill their daily tasks and needs that cannot be done without it. It is estimated that about 48 percent of the world’s population use the Internet [1,2]. This means half of the population use the Internet due to its popularity and benefits that are provided to the people by the Internet. Another aspect of increasing the users of the Internet is that people can communicate and synchronize to other people all over the world via the Internet.

Due to the benefits of the Internet, another field is growing, which allows objects and machines to connect and communicate to each other with the presence of the Internet, called the Internet of Things (IoT) [3]. The concept behind this new technology is to automate the work and connect the devices via the Internet that we use in our daily life. Special types of sensors are attached to each object to capture the information from the physical world. Information is analyzed by local processing to remove the unnecessary data and store the information into local storage. Information is sent from local storage to cloud storage where all objects send their collected information. Finally, using the gathered information, an appropriate action is taken. It is not compulsory that action is always performed by using this information, but we can also manage and control the objects and machines remotely and use the information to maintain the records for the future use.

There are several technologies and sensors used to implement the idea of IoT. The communication technologies which are used to implement the idea of IoT are radio frequency identification (RFID) [4], near field communication (NFC) [5] and wireless sensor network (WSN) [6], etc.

**Figure 1:**
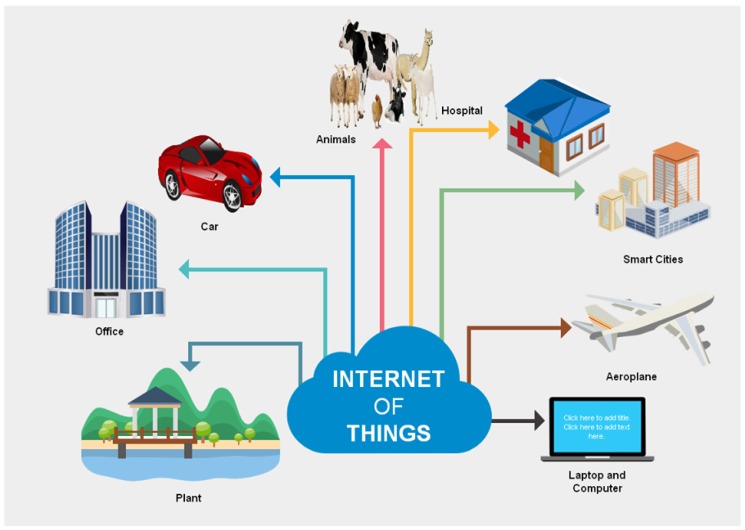
Application domains of IoT.

There are a lot of applications in which IoT has deployed as shown in Figure 1. They have become smart and perform their work robotically by taking help from the Internet [7,8]. The first one is the health care domain where sensors are used to check human’s body temperature, blood pressure and heart beat rate [9]. Another application is smart home because humans use many electronic things like refrigerators, microwave ovens, fans, heaters and air conditioners at home. The sensors are installed to detect the problem and tell about the problem to the manufacturing company in order to solve it [10]. The third application of IoT is animal tracking. The GPS sensors are installed in an animal’s body to trace them easily. It is also used to monitor the animal’s feed [11]. Another IoT application is smart robotics grippers that contact an object directly to collect the sensing information. There are a lot of sensors and instruments installed in a smart gripper such as touch, motion, vision, optical and force sensors. The smartness level of a smart gripper depends on the equipped sensors because they collect information in a real-time mode and collected information is used to make decisions. Therefore, they must be confined by design criteria such as cost, weight and compactness [12]. In addition, there are numerous applications of IoT such as smart transportation, infrastructure management (highways, bridges and railway tracks), manufacturing, smart building, smart agriculture and smart retail, etc. Table 1 shows the application domains of IoT. Furthermore, it also compares the different application domains with respect to the number of users, communication technology, network size, bandwidth and their test beds.

**Figure 2:**
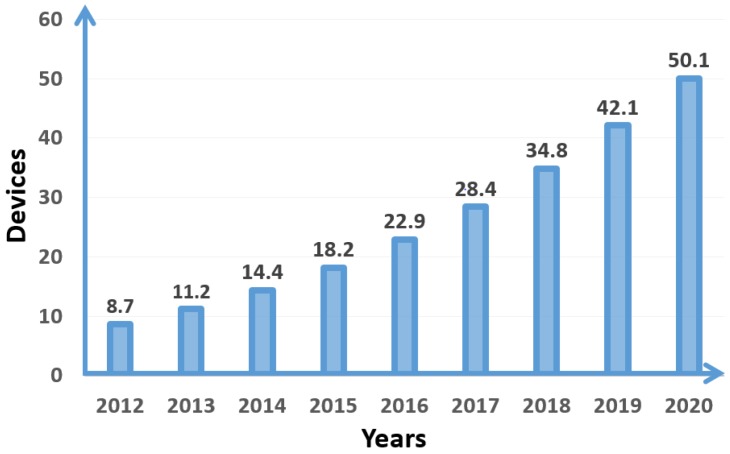
Number of connected IoT devices from 2012 to 2020.

The number of IoT devices are increasing each and every day. The reason for increasing the number of IoT devices is that they provide comfort in human life and perform work with better outcomes than humans. It has been reported that, in 2018, the number of IoT devices will have more than tripled since 2012 and there will be 50 billion devices that will work on the Internet [18]. Figure 2 shows the number of connected IoT devices from 2012 to 2020.

People use the new technology named Internet of Thing (IoT) not only due to popularity but also the benefits and services provided by it. With IoT, tasks can be performed without using human collaboration so it makes the life simple and easy. It allows the people to automate, achieve and control the tasks that are essential for life and provide better responses as a result.

There are many benefits provided by IoT, but, on the other side, it also has some challenges such as poor management, energy efficiency, identity management, security and privacy [19]. Security and privacy are the most critical issues facing in the development of IoT. In IoT, all devices are connected to the Internet because, without the Internet, they cannot perform their tasks. There are many attackers on the Internet that steal the confidential information of objects. The attackers can use information of users in any illegal way according to their needs, which can result in a great loss for users [20]. Privacy has also become an issue for IoT. This means that the information of users must be in secure hands and not be accessible to anyone except authentic users. Therefore, it has bigger scope than security [21]. Therefore, security and privacy should be ensured by preventing unauthorized identification, access and user’s data is under his control and no one else’s.

The objectives of this paper are manifold:

- Give a picture of all proposed layered architecture of IoT,
- Highlight the security attacks that can occur on each layer and affect the IoT applications,
- Present the communication technologies used by IoT applications along with characteristics and drawbacks as well,
- Provide information about security mechanisms used to protect IoT.
- Suggest a new and generic six-layered secure architecture that can easily be extended with little impact to existing architectures to make secure IoT applications.

The rest of the paper is organized as follows: Section 2 describes the basic elements of IoT used to perform its works. Section 3 discusses the different opinions about layer architectures of IoT along security threats and problems faced by IoT layers. Section 4 provides an overview of communication technologies of IoT with their characteristics and drawbacks. Section 5 provides an overview of current security mechanisms to protect the IoT with their limitations. A new and generic secure layered architecture is suggested in Section 6. Section 7 describes the research challenges and also future direction in the security field of IoT. Furthermore, Section 8 describes the conclusions of this survey paper.

## 2. IoT Elements

**Figure 3:**
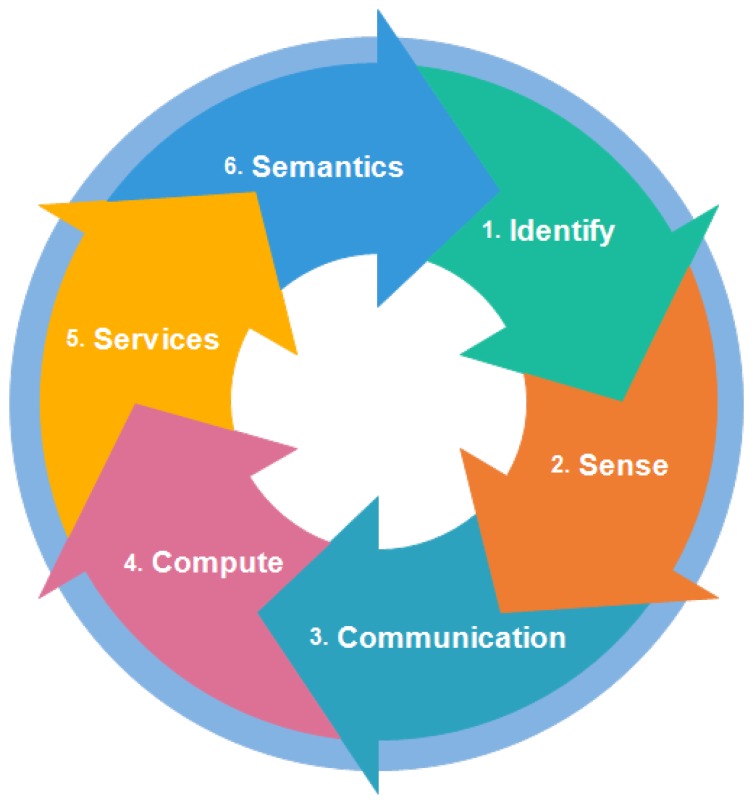
The IoT elements.

IoT provides many benefits and facilities to users. Thus, in order to use them properly, there is a need for some elements. In this section, elements of IoT are discussed. Figure 3 shows the elements needed to deliver the functionality of IoT. The names and details of these elements are as follows.

### 2.1. Identification

Identification offer explicit identity for each object within network. There are two processes in identification; naming and addressing. Naming refers as name of the object while addressing is the unique address of specific object. These both terms are very different from each other because two or more objects may have same name but always different and unique address. There are many methods available that provide the naming facility to the objects in the network such as electron products codes (EPC) and ubiquitous codes [22]. To assign the unique address to each object, IPv6 is used. Firstly, IPv4 was used to assign the address but it could not fulfill the need of addressing due to large amount of IoT devices. Therefore, IPv6 is used because it uses 128 bit number addressing scheme.

### 2.2. Sensing

The process of collecting information from objects is known as sensing. The collected information is sent to the storage media. There are many sensing devices to collect the information from objects such as actuators, RFID tags, smart sensors, wearable sensing devices, etc.

### 2.3. Communication

Communication is one of the main purposes of IoT in which different devices are connected to each other and communicate. In communication, devices may send and receive messages, files and other information. There are many technologies that provide facility of communication like Radio Frequency Identification (RFID) [23], Near Field Communication (NFC) [24], Bluetooth [25], Wi-Fi [26] and Long Term Evolution (LTE) [27].

### 2.4. Computation

Computation is performed on the collected information from the objects by using sensors. It is used to remove unnecessary information that is not needed. Many hardware and software platforms are developed to perform the processing in applications of IoT. For hardware platforms, Audrino, Rasperry Pi and Intel Galileo are used, while, for software platforms, the operating system plays an important role to perform the processing. There are many types of operating systems that are used like Tiny OS [28], Lite OS [29], Android, etc.

### 2.5. Services

There are four types of services that are provided by the IoT applications [30,31]. The first one is an identity-related service. It is used to get the identity of objects that have sent the request. Information aggregation is another service whose purpose is to collect all the information from objects. Processing is also performed by the aggregation service. The third service is a collaborative service that makes decisions according to the collected information and sends appropriate responses to the devices. The last service is ubiquitous service, which is used to respond the devices immediately without rigidity about time and place.

### 2.6. Semantics

It is the responsibility of IoT to facilitate users by performing their tasks. It is the most important element of IoT to fulfill its responsibilities. It acts like the brain of IoT. It gets all information and makes appropriate decisions to send responses to the devices.

Table 2 is shown to describe the key technologies involved in each element of IoT.

## 3. IoT Layered Architectures with Security Attacks

There is no single and general agreement about the architecture of IoT that is agreed on by the whole world and researchers. Many and different architectures have been proposed by researchers. According to some researchers, IoT architecture has three layers, but some researchers support the four-layer architecture. They think that, due to enhancement in IoT, the architecture of three layers cannot fulfill the requirements of applications. Due to a challenge in IoT regarding security and privacy, the architecture of five layers has also been proposed. It is considered that a recently proposed architecture can fulfill the requirements of IoT regarding security and privacy.

### 3.1. Three Layer Architecture

**Figure 4:**
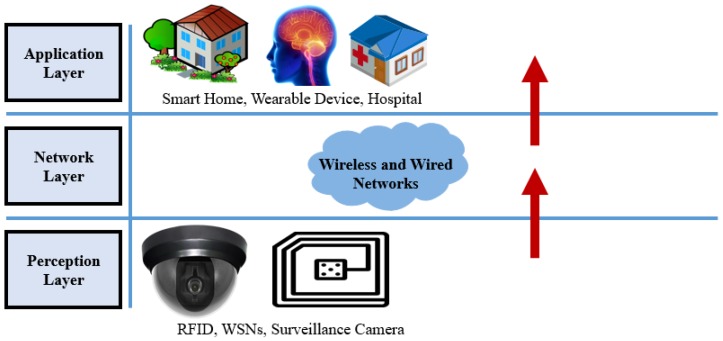
The three-layered architecture of IoT.

It is a very basic architecture and fulfills the basic idea of IoT. It was proposed in the early stages of development of IoT [32,33,34]. It has three layers. The names of these three layers are perception, network and application layer as shown in Figure 4.

#### 3.1.1. Perception Layer

It is also known as a sensor layer. It works like people’s eyes, ears and nose. It has the responsibility to identify things and collect the information from them. There are many types of sensors attached to objects to collect information such as RFID, 2-D barcode and sensors. The sensors are chosen according to the requirement of applications. The information that is collected by these sensors can be about location, changes in the air, environment, motion, vibration, etc. However, they are the main target of attackers who wish to utilize them to replace the sensor with their own. Therefore, the majority of threats are related to sensors [35,36,37]. Common security threats of perception layer are:

- Eavesdropping: Eavesdropping is an unauthorized real-time attack where private communications, such as phone calls, text messages, fax transmissions or video conferences are intercepted by an attacker. It tries to steal information that is transmitted over a network. It takes advantage of unsecure transmission to access the information being sent and received.
- Node Capture: It is one of the hazardous attacks faced in the perception layer of IoT. An attacker gains full control over a key node, such as a gateway node. It may leak all information including communication between sender and receiver, a key used to make secure communication and information stored in memory [38].
- Fake Node and Malicious: It is an attack in which an attacker adds a node to the system and inputs fake data. It aims to stop transmitting real information. A node added by an attacker consumes precious energy of real nodes and potentially control in order to destroy the network.
- Replay Attack: It is also known as a play back attack. It is an attack in which an intruder eavesdrops on the conservation between sender and receiver and takes authentic information from the sender. An intruder sends same authenticated information to the victim that had already been received in his communication by showing proof of his identity and authenticity. The message is in encrypted form, so the receiver may treat it as a correct request and take action desired by the intruder [39].
- Timing Attack: It is usually used in devices that have weak computing capabilities. It enables an attacker to discover vulnerabilities and extract secrets maintained in the security of a system by observing how long it takes the system to respond to different queries, input or cryptographic algorithms [40].

#### 3.1.2. Network Layer

Network layer is also known as transmission layer. It acts like a bridge between perception layer and application layer. It carries and transmits the information collected from the physical objects through sensors. The medium for the transmission can be wireless or wire based. It also takes the responsibility for connecting the smart things, network devices and networks to each other. Therefore, it is highly sensitive to attacks from the side of attackers. It has prominent security issues regarding integrity and authentication of information that is being transported in the network. Common security threats and problems to network layers are:

- Denial of Service (DoS) Attack: A DoS attack is an attack to prevent authentic users from accessing devices or other network resources. It is typically accomplished by flooding the targeted devices or network resources with redundant requests in an order to make it impossible or difficult for some or all authentic users to use them [41].
- Main-in-The-Middle (MiTM) Attack: MiTM attack is an attack where the attacker secretly intercepts and alters the communication between sender and receiver who believe they are directly communicating with each other. Since an attacker controls the communication, therefore he or she can change messages according to their needs. It causes a serious threat to online security because they give the attacker the facility to capture and manipulate information in real time [42].
- Storage Attack: The information of users is stored on storage devices or the cloud. Both storage devices and cloud can be attacked by the attacker and user’s information may be changed to incorrect details. The replication of information associated with the access of other information by different types of people provides more chances for attacks.
- Exploit Attack: An exploit is any immoral or illegal attack in a form of software, chunks of data or a sequence of commands. It takes advantage of security vulnerabilities in an application, system or hardware. It usually comes with the aim of gaining control of the system and steals information stored on a network [43].

#### 3.1.3. Application Layer

Application layer defines all applications that use the IoT technology or in which IoT has deployed. The applications of IoT can be smart homes, smart cities, smart health, animal tracking, etc. It has the responsibility to provide the services to the applications. The services may be varying for each application because services depend on the information that is collected by sensors. There are many issues in the application layer in which security is the key issue. In particular, when IoT is used in order to make a smart home, it introduces many threats and vulnerabilities from the inside and outside. To implement strong security in an IoT based smart home, one of the main issues is that the devices used in smart homes have weak computational power and a low amount of storage such as ZigBee [44]. Common security threats and problem of application layer are:

- Cross Site Scripting: It is an injection attack. It enables an attacker to insert a client-side script, such as java script in a trusted site viewed other users. By doing so, an attacker can completely change the contents of the application according to his needs and use original information in an illegal way [45].
- Malicious Code Attack: It is a code in any part of software intended to cause undesired effects and damage to the system. It is a type of threat that may not be blocked or controlled by the use of anti-virus tools. It can either activate itself or be like a program requiring a user’s attention to perform an action.
- The ability of dealing with Mass Data: Due to a large number of devices and a massive amount of data transmission between users, it has no ability to deal with data processing according to the requirements. As a result, it leads to network disturbance and data loss.

### 3.2. Four Layer Architecture

**Figure 5:**
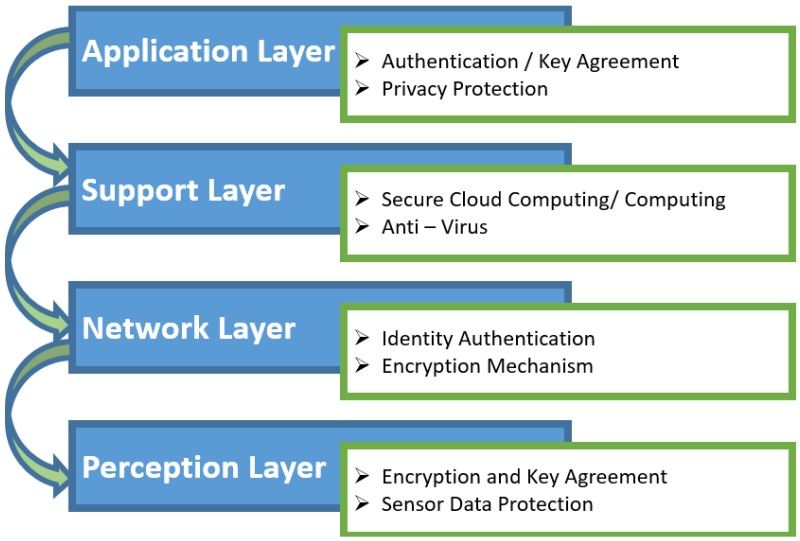
The four-layered architecture of IoT along recommended security mechanisms.

The three-layer architecture was most basic architecture. Due to continuous development in IoT, it could not fulfill all the requirements of IoT. Therefore, researchers proposed an architecture with four layers [46]. It has three layers like the previous architecture, but it also has one more layer called a support layer. Figure 5 presents the layered architecture of it along recommended security mechanisms used to make it secure from intruders. The three layers have the same functionality as the three-layer architecture that we have already discussed above so the functionality of the support layer along security attacks is as follows:

#### Support Layer

The reason to make a fourth layer is the security in architecture of IoT. Information is sent directly to the network layer in three-layer architecture. Due to sending information directly to the network layer, the chances of getting threats increase. Due to flaws that were available in three-layer architecture, a new layer is proposed. In four-layer architecture, information is sent to a support layer that is obtained from a perception layer. The support layer has two responsibilities. It confirms that information is sent by the authentic users and protected from threats. There are many ways to verify the users and the information. The most commonly used method is the authentication. It is implemented by using pre-shared secrets, keys and passwords. The second responsibility of the support layer is sending information to the network layer. The medium to transmit information from the support layer to network layer can be wireless and wire based. There are various attacks that can affect this layer such as DoS attack, malicious insider, unauthorized access, etc. Common threats and problems of the support layer are:

- DoS Attack: The DoS attack in a support layer is related to the network layer. An attacker sends a large amount of data to make network traffic inundated. Thus, the massive consumption of system resources exhausts the IoT and makes the user not capable of accessing the system.
- Malicious Insider Attack: It occurs from the inside of an IoT environment to access the personal information of users. It is performed by an authorized user to access the information of other user. It is a very different and complex attack that requires different mechanisms to prevent the threat [47,48].

### 3.3. Five Layer Architecture

The four-layer architecture played an important role in the development of IoT. There were also some issues regarding security and storage in four-layer architecture. Researchers proposed five-layer architecture to make the IoT secure [49,50,51]. It has three layers like previous architectures whose names are perception layer, transport layer and application layer. It also has two more layers. The names of these newly proposed layers are processing layer and business layer. It is considered that the newly proposed architecture has the ability to fulfill requirements of IoT. It also has the ability to make the applications of IoT secure. The workings of these layers and security attacks that can effect them are as follows:

#### 3.3.1. Processing Layer

The processing layer is also known as a middleware layer. It collects the information that is sent from a transport layer. It performs processing onto the collected information. It has the responsibility to eliminate extra information that has no meaning and extracts the useful information. However, it also removes the problem of big data in IoT. In big data, a large amount of information is received which can affect performance of IoT. There are numerous attacks that can affect the processing layer and disturb the performance of IoT. Common attacks are:

- Exhaustion: An attacker uses exhaustion to disturb the processing of IoT structure. It occurs as an after-effect of attacks, such as DoS attack in which an attacker sends the victim many requests to make the network unavailable for users. It could be a result of other attacks that aim to exhaust the system resources, such as battery and memory resources. IoT has a distributed nature; therefore, it does not have a high amount of hazards. It is much easier to implement protecting procedures against it [52].
- Malwares: It is an attack on the confidentiality of the information of users. It refers to the application of viruses, spyware, adware, Trojans horses and worms to interact with the system. It takes the form of executable codes, scripts and contents. It acts against the requirements of system to steal the confidentially of information [53].

#### 3.3.2. Business Layer

The business layer refers to an intended behavior of an application and acts like a manager of a whole system. It has responsibilities to manage and control applications, business and profits models of IoT. The user’s privacy is also managed by this layer. It also has the ability to determine how information can be created, stored and changed. Vulnerability in this layer permits the attackers to misuse an application by avoiding the business logic. Most problems regarding security are weaknesses in an application that result from a broken or missing security control. Common problems regarding security of business layer are:

- Business Logic Attack: It takes advantage of a flaw in a programming. It controls and manages the exchange of information between a user and a supporting database of an application. There are several common flaws in the business layer, such as improper coding by a programmer, password recovery validation, input validation, and encryption techniques [54].
- Zero-Day Attack: It refers to a security hole or a problem in an application that is unfamiliar to a vendor. This security hole is exploited by the attacker to take control without user’s consent and without their knowledge [55,56].

**Figure 6:**
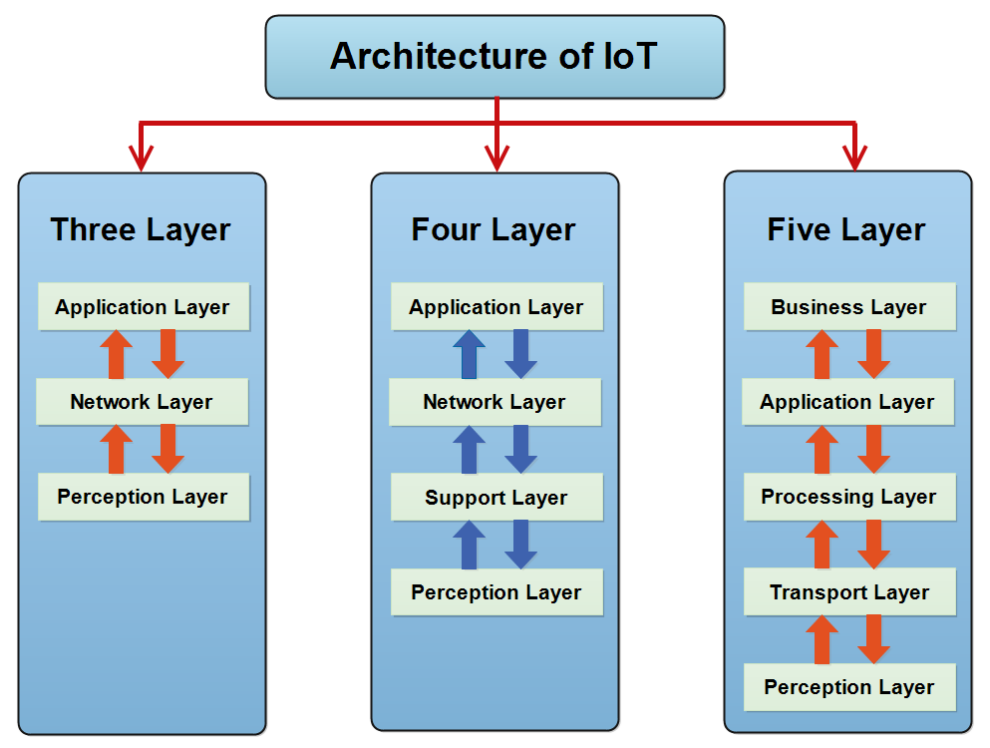
The layered architectures of IoT (three, four and five layers).

The hierarchy of all proposed layered architecture of Internet of Things (IoT) is shown in Figure 6, which shows the layer architectures of IoT consisting of three layers, four layers and five layers respectively.

## 4. Security Issues in Communication Technologies of IoT

Each technology has some security features and also provides the security protocols that are used to communicate. The characteristics as well as drawbacks of these technologies and applications are as below:

### 4.1. ZigBee Technology

**Figure 7:**
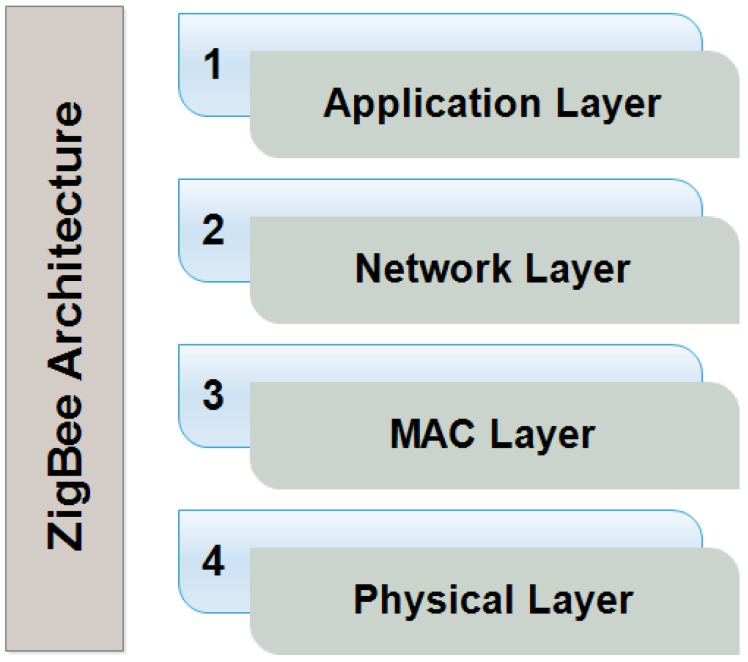
The architectures of ZigBee.

ZigBee [57,58] is a PAN (personal area network). It provides low-power consumption at a low cost to obtain the trust of maximum users. It provides wireless communication to transmit information within a short range. The layered architecture of ZigBee technology is drawn in Figure 7 consisting of four layers by named application, network, Media Access Control (MAC) and physical layer.

The MAC layer is used to provide security in ZigBee technology. There are three types of services to provide security. The first one is access control. It is a security technique that can be used to control and manage who or what can view or use resources in a system. The second service is encryption, which is provided by a MAC layer. It provides the facility to change the message into another form, called cipher text, which cannot be understood by anyone except authorized users. Integrity is also provided by the third layer of ZigBee. The most common attack is man-in-the-middle attack. It receives information from a sender and makes changes in it. The MAC layer provides facility of integrity as a third service to control a man-in-the middle attack. It provides the ability for the receiver to check contents of messages that have been changed or modified from attackers.

ZigBee technology provides security by assigning a network key to each device. It is compulsory for each device to have a network key that is assigned at the time of registration. When the device sends a request for communication, the network key is asked from it. Therefore, only authorized and authentic devices can communicate to each other. It also has some drawbacks. The network key that is assigned can never be changed. It does not provide any facility to update or change the key, which is not a good tactic regarding security.

There are many applications of IoT in which ZigBee technology is used like a home energy monitoring and control system [59], a fingerprint based attendance system [60], and a greenhouse monitoring system [61].

### 4.2. Bluetooth Technology

Bluetooth [62] is used for applications that want to communicate within a short distance. It provides many security mechanisms to make secure communication between sender and receiver. It provides a facility of encryption in which a message is converted into another form, called cipher text. On the other side, the receiver also has the ability to change cipher text into an original message. Due to encryption, a message cannot be understood by anyone except the user who has the rights to see the message. The sender must get permission from the receiver before sending the message. First of all, a request is sent to a receiver that has information that the sender wants to share. It depends on the receiver to accept or reject the sender’s request.

There are many threats that can affect performance of Bluetooth. The most common threat that is faced by the Bluetooth technology is blue jacking. It is usually harmless because users generally do not know what has happened. It sends a text message, but, with a modern phone, it is possible to send images or sounds as well. The second threat is bluesnarfing. It is unauthorized access of information. It allows access to calendars, contact lists, email and text messages. It can also copy user’s images and private videos. The difference between both of these threats is that blue jacking is essentially harmful as it only transmits data to the target device while bluesnarfing is theft of information from the target device.

There are many applications of IoT in which Bluetooth technology is used like human traces [63], smart home automation [64] and a traffic monitoring system [65].

### 4.3. Radio Frequency Identification

RFID [66] uses frequency waves for communication between two devices. It has three parts: tags, reader and a database. The tags are attached to the objects and read the state of the objects while a reader is used to read the information from tags. A database is considered as a third part and it is used to store information.

It provides the facility of encryption to transmit the information. There are three types of encryption that can be used by it. In the first type, encryption is not used. It is not used to send unnecessary information. The aim is to save resources of the system. The second type of encryption is data encryption standard (DES). It is a symmetric-key method of data encryption. It uses the same key to encrypt and decrypt a message. Therefore, both the sender and receiver must know and use a private key. The third type is advance encryption standard (AES). It is a more efficient and elegant cryptographic algorithm. Its main strong point rests in key length options. The time required to break an encryption algorithm is directly related to the length of the key. It allows choosing a 128-bit, 192-bit or 256-bit key to create more strength than the 56-bit key of DES. Therefore, it is considered secure than DES.

It provides the facility of encryption to make the information secure, but it also has some drawbacks. It does not provide security to read information from tags because tags give information without verifying the authentication of reader. The attacker can make his own reader to collect information. There should be a mechanism to verify authentication of a reader before giving information to a reader. It also has a problem regarding truthfulness in which an attacker can easily change the behavior of tags according to his needs. It provides encryption in which contents of a message is converted into an unknown form, called cipher text. Due to development in information technology (IT), attackers can change cipher text. Therefore, integrity has become an issue in which a receiver does not know about the changes [67].

There are many applications of IoT in which RFID technology is used, such as a healthcare system [68], human tracking application [69] and a gesture recognition system [70].

### 4.4. Wireless Sensor Network

**Figure 8:**
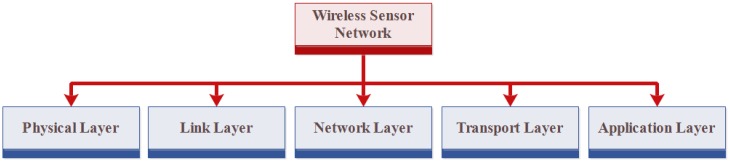
The layered architecture of a wireless sensor network.

WSNs have many nodes and each node has four parts: sensors, battery, microcontroller and memory. The functionality of WSN can easily be understood in this way in which sensors are used to collect the information and store it in its memory for reuse. It sends all information to the server. Batteries are also used that provide facilities to work continuously. Smart grid [71], environmental monitoring [72] and an intrusion detection system [73,74] are the applications of IoT in which a wireless sensors network is used. The architecture of WSN consists of five layers, named physical, link, network, transport and application layer, as shown in Figure 8.

There are several attacks in WSN, such as service attacks, authentication problem, Denial of Service (DOS) and Distributed DOS (DDOS) [75].

### 4.5. Wireless Fidelity (Wi-Fi)

Wi-Fi is a wireless communication network that transmits communication in the form of radio signals. There are few issues regarding security in it. It does not provide an encryption mechanism. Therefore, it is easy to change the message by the attacker. The second drawback is eavesdropping. It refers to the unauthorized observing and monitoring of other’s people communication. It is a process of gathering the information of users transmitted over a network [76].

There are many applications in which Wi-Fi technology is used, such as indoor positioning system [77,78], smart home implementation system [79] and rehabilitation system [80].

### 4.6. 5G Networks

There are many communication technologies such as 2G/3G/4G, ZigBee, Bluetooth, RFID and WSN (discussed above) used in IoT applications. These communication technologies face a lot of challenges due to a large number of connecting devices, security threads, new standards, device computational capabilities and complicated communication [81]. To address these challenges, the next generation network, named a 5G network, is becoming more rapidly available with IoT that fulfills the needs of smart environment and industry [82]. It is the next (fifth) generation of cellular technology that promises to enhance speed, coverage and responsiveness of wireless network. Due to consideration of both the research community and academia on 5G based IoT, it is reported that full 5G will be available after 2025 [83]. There are lot of requirements of IoT applications such as high data rates, high scalability, low latency, reliability, security, high battery lifetime and mobility, provided by the 5G network. However, it can satisfy requirements of IoT, but, on the other hand, it opens a set of challenges on the architecture of 5G-IoT. The challenges that need to be considered are: scalability, heterogeneity, security assurance and privacy concerns [84].

The name of all communications technologies, their communication mechanisms, distance range covered by them, security mechanisms, applications, characteristics and also the drawbacks are shown in Table 3.

## 5. Security Mechanisms for IoT

**Figure 9:**
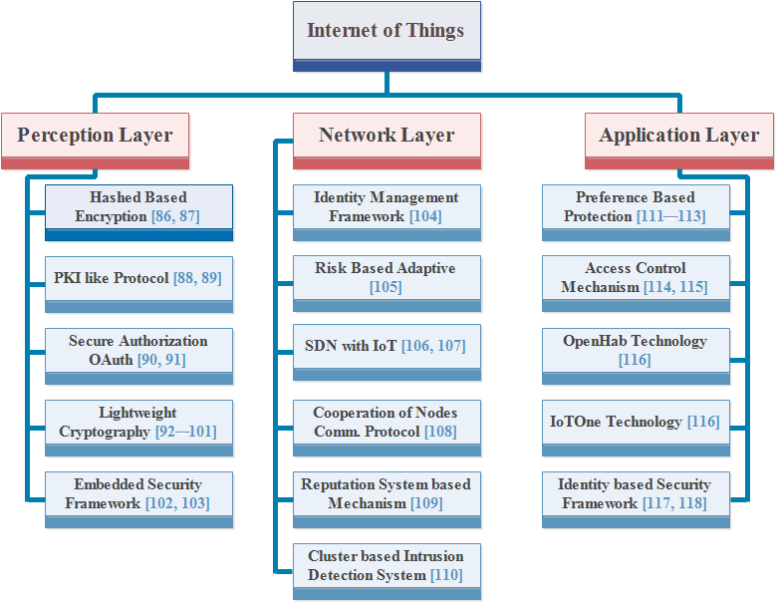
Existing security mechanisms to protect the IoT applications.

Security is a critical issue that exists in IoT. We cannot use the IoT properly and cannot be able to attain all the benefits that are provided by IoT without security [85]. In the following, we present prominent security mechanisms that have been proposed in the literature. Figure 9 shows the existing security mechanisms used to protect the IoT applications from intruders.

### 5.1. Encryption and Hashed Based Security

Internet is the most important part of IoT. The information passes through a network during the communication where attackers also exist. Therefore, user’s information is not secured on the network. There should be a mechanism to protect information from the attackers. To make user’s information secure, a researcher proposed a method whose name is encryption and hashed based security. It provides a facility of encryption in which a message is converted into an unknown form, called cipher text. When message is sent from a sender, it is converted into another form by using a key that cannot be understood to anyone except authentic users. It generates a key according to the length of the message. It always has a key of double length from the message. Therefore, it is not easy task to break a key. The key is also forwarded to the receiver. The receiver has the ability to covert the cipher text into an original message by using the key. This method greatly helps with making the user’s information secure, but, due to development in information technology, it is possible to change the contents of cipher text for the attackers. The attacker tries to make a message corrupt for the receiver. Therefore, this method also provides a facility of hash function. It is used to know and recover the contents of the message that have been changed by the attacker. It is used along with encryption. It provides a digital fingerprint and a digital watermark of a message’s contents, which ensures that a message has not been altered by an intruder, virus, or by other means [86,87].

### 5.2. Public Key Infrastructure (PKI) Like Protocol

Many mechanisms are suggested or proposed to eliminate the issue of security. In the encryption method, a sender changes the message into another form that cannot be understood by anyone except the receiver. The encryption is performed by using a key and forwarded to a receiver through a message.

The receiver converts a message by taking help of a key. Thus, the method makes messages secure from the attackers. The second proposed method is authorization and authentication to save the messages from intruders. Authorization is a security mechanism used to determine user and client rights and access levels related to system resources. It includes computer programs, files, data, services and application features. Authentication is a process that permits a device to confirm the identity of someone who connects to a network resource. Authorization is normally preceded by authentication. Intrusion detection is also considered as a security mechanism. It monitors and controls activities of the network to see their behavior. If there exist some changes in the environment, it performs some countermeasurements to stop the illegal activity immediately [88]. These mechanisms are implemented in different layers of IoT to save information from attackers. We cannot say that the security of IoT can be improved by adopting one method. To make it secure, we should use more than one measure.

A Public Key Infrastructure (PKI) like protocol mechanism is a combination of all the mechanisms described above. It is implemented in the recognition layer of IoT architecture. It is better than using different mechanisms individually. There are many nodes connected to each other and they make a network. It has a responsibility to provide security. Therefore, it does not trust anyone to send a message.

**Figure 10:**
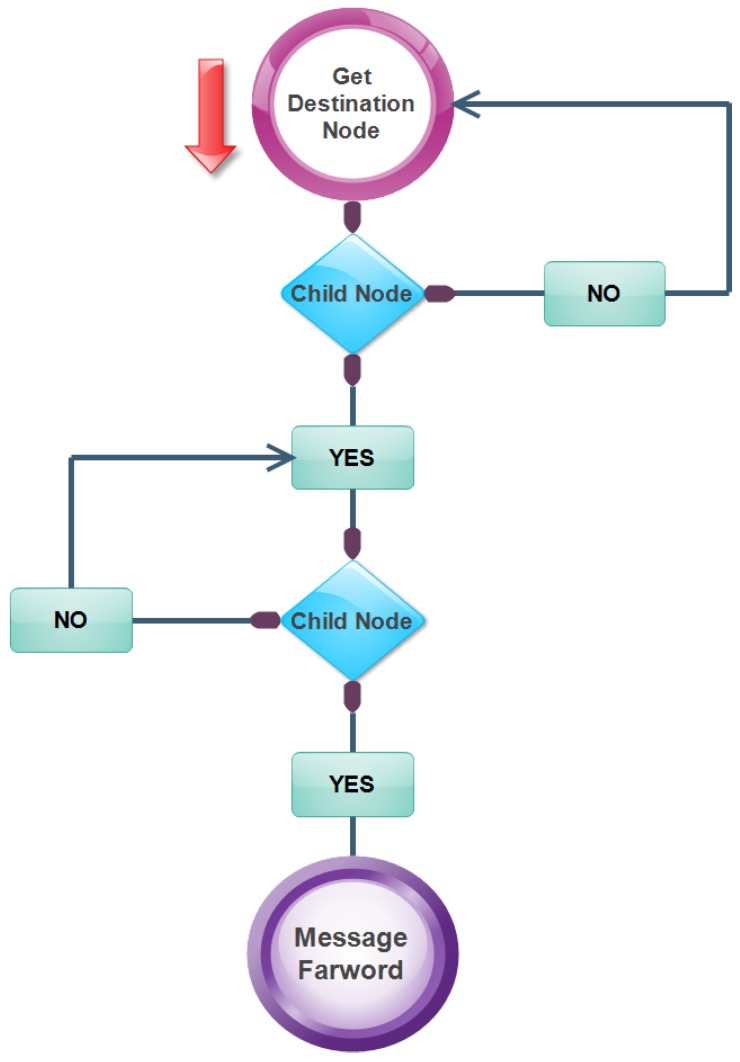
The PKI like protocol for IoT.

The working of PKI like protocol can be understood in this way that the nodes are available in a form of tree in the network. The root node acts like a base station in the tree. It uses an RSA (Rivest–Shamir–Adleman) encryption algorithm as the public key and privacy key, respectively. The public key is stored at the base station while the privacy key is distributed to each node by a base station. Figure 10 is used to illustrate the working of it. When the message is sent from the sender to the receiver node, it is transmitted to the child node of a receiver node. The child node further sends a message to the other child node. This process persists unless a message’s receiver is found. The former node is required to check the authenticity of the other node before sending the message. If the receiver node is found in the same network, then a message should be transmitted directly. Conversely, if the receiver node is not found in the network, the message is sent to the base station. It broadcasts the public key to the whole network and finds the receiver node from other networks. Afterwards, the message is transmitted to the receiver node by using child nodes of the receiver node [89].

### 5.3. Secure Authorization Mechanism with OAuth (Open Authorization)

To implement the authorization mechanism, three questions must be addressed, which are:

- Which users have rights to access the specific information?
- What should be a mechanism to access the services?
- Which types of operation that can be performed by the users?

There are two terms that are used in authorization mechanism, which are: Role Based Access Control (RBAC) and Attributes Based Access Control (ABAC). RBAC permits those users who have rights to use it; otherwise, it will not provide permission to any other to use a specific service, while ABAC permits specific attributes that are assigned to the authorized user.

The problem that exists in the authorization mechanisms is that third party organization can access the user’s information. There are many ways to access the user’s information. For example, an attacker can easily access the information by showing itself as a real user to the service provider. The reason behind this is that credentials are not managed by the users. To solve this problem, OAuth (open authorization) protocol is proposed [90]. It has four characters through which communication between clients and server become possible which are; owner, server (service provider), client and authorization server.

**Figure 11:**
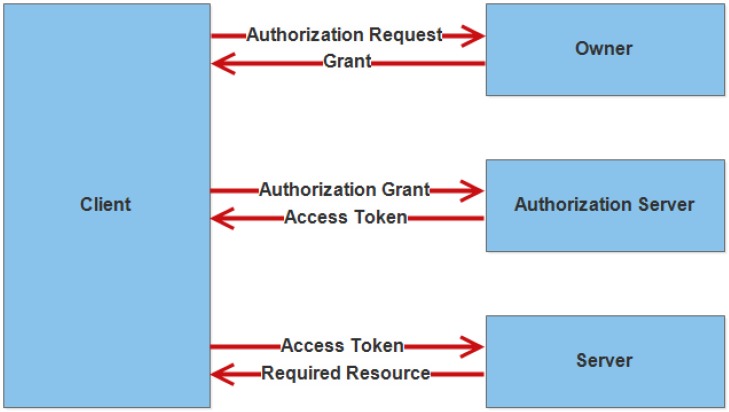
IP based open authorization system for IoT.

Figure 11 shows the character and its roles regarding OAuth mechanism.

The working of OAuth can easily be understood in this way in which a client sends a request to the owner. The request can be sent directly or indirectly by the client. The authorization grant is provided to the client. The authorization server provides an access token after receiving an authorization grant. At the end, control goes to the server. It takes an access token from the client and provides the required resource to the client [91].

### 5.4. Lightweight Cryptographic Algorithms

Lightweight cryptography is a mechanism that is used to meet the security requirements in smart devices [92]. There are three types of lightweight cryptography mechanisms whose names are symmetric key lightweight cryptographic algorithm, public key lightweight cryptographic algorithm and hash functions. These are used according to the requirements of users and messages that have to be transmitted. The details of these algorithms are as under:

#### 5.4.1. Symmetric Key Lightweight Cryptographic Algorithm

It provides an encryption system in which the sender and receiver of a message share a single and common key. It is used to convert the message by a sender into an unknown form, called cipher text. It is also used to convert the message by a receiver from cipher text to the original message. Thus, the message can only be understood by authentic users.

There are various examples of symmetric key cryptography algorithm such as Blowfish, Advanced Encryption Standard (AES), Data Encryption Standard (DES) and Rivest Cipher or Ron’s Code, but the most widely used algorithm is AES. The AES encryption algorithm has become the optimal choice in order to provide security as well as improve efficiency and performance for numerous applications [93]. The work presented in [94] proposes a distributed system in order to make data communication of the whole network secure. The communication occurs in two segments: IoT devices to the IoT gateways and IoT gateways to the Internet. The communication in these segments is secured by applying a symmetric key cryptography algorithm by AES. The main disadvantage of this encryption scheme is that both parties (sender and receiver) have to exchange the key used to encrypt the data between them before they can decrypt it.

#### 5.4.2. Public Key Lightweight Cryptographic Algorithm

It is also known as an asymmetric lightweight cryptographic algorithm. It demands use of both keys: a public key and a private key. A public key converts the messages into cipher text while a private key decrypts them. A Public key represents unique identification of a node that is provided by the certification authority (CA). It requires high processing, high energy consumption and long keys rather than symmetric key encryption. It has two types: RSA (Rivest, Shamir, and Adleman) and ECC (Elliptic Curve Cryptography). The RSA needs a 1024-bits long key to encrypt the messages [95]. ECC is more effective than RSA. It demands less bits to encrypt the messages than RSA [96]. There is no difference between the RSA and ECC in terms of security because both encryptions provide the same security levels. Therefore, ECC is considered better and more effective than RSA while having low processing and low energy consumption. This type of encryption is used by many organizations like American National Standards Institute (ANSI) [97], Institute of Electrical and Electronics Engineers (IEEE) [98], International Standards Organization (ISO) [99], Standards for Efficient Cryptography Group (SECG) [100] and National Institute of Standards and Technology (NIST) [101].

#### 5.4.3. Cryptographic Hash Functions

Hash function plays a different role than other cryptographic algorithms. Hash functions are usually used in many aspects of security, such as digital signature and data integrity checks. They take messages, blocks of data or electronic files and generate a digital fingerprint of the contents, called a hash value. The key property of a hash function is that when an attacker changes the input, then it affects the output. Thus, the receiver finds changes in the message.

The secret key must exchange before using an encryption method. Distribution of secret keys has been problematic until recently. It included face-to-face meetings, use of a trusted messenger or sending the key through an existing channel. The first two are always unsafe while the third depends on the security of a previous key exchange. There are many key agreement protocols that can be used for distribution like polynomial based key distribution protocol, a possible alternative protocol, etc.

### 5.5. Embedded Security Framework

There are many attacks that not only affect the system but also gain the information of the users. Physical attack directly affects the hardware components of the system, but these attacks are not used practically due to cost. A cryptanalysis attack refers to the study of cipher text to find a weakness that will permit recovery of the original message from cipher text, without knowing the key. A software attack is in form of file or program. The attacker sends a program or file to harm a computer user. These attacks can perform a variety of functions, including stealing, deleting sensitive information, and monitoring user’s activities without their permission. Network attack increases the traffic by adding fake nodes to make network unavailable for the users. Man-in-the-middle attack is a type of network attack. The attacker secretly relays and modifies the communication between two parties who trust that they are directly communicating with each other.

**Figure 12:**
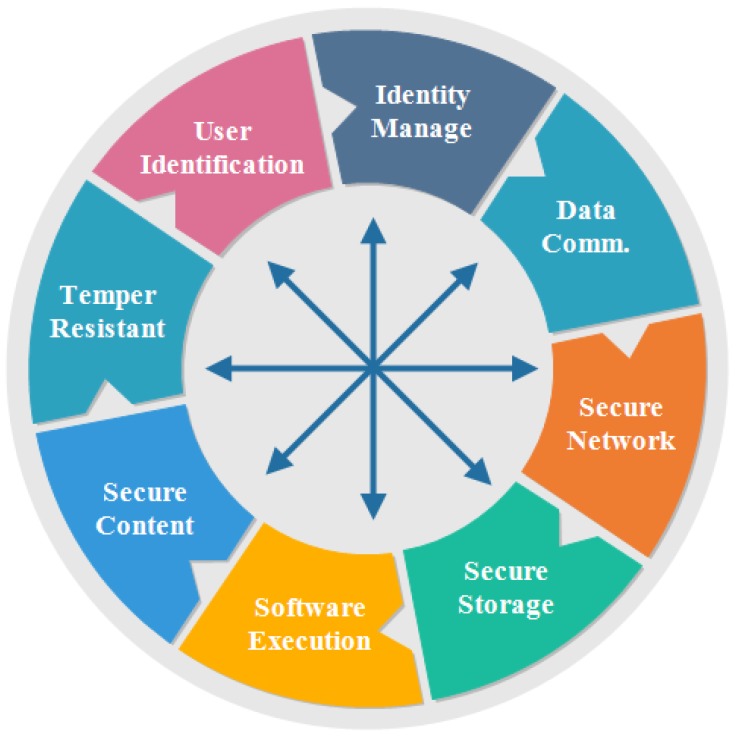
The key security concerns in IoT.

Figure 12 lists the typical security requirements seen across an extensive range of IoT to prevent from attacks [102]. The security requirements are described as follows:

#### 5.5.1. User Identification

It refers to the process of authenticating users before permitting them to use the resources of the system. There are many ways that can be used to validate users, including pre-shared key or password.

#### 5.5.2. Identity Management

It deals with identifying individual device in a system. It also controls their access to resources within that system by associating user rights and limitations with the recognized entity.

#### 5.5.3. Secure Data Communication

It demands authentication of communicating devices, make sure confidentiality and integrity of communicated data and protecting the entities of communicating devices.

#### 5.5.4. Secure Network

Secure network access provides a network connection or service only if the device is authorized. The unauthorized devices are not allowed to access the network.

#### 5.5.5. Secure Storage

Secure storage has a responsibility to save the information of users from intruders and external monitoring. Therefore, it mandates confidentiality and reliability of sensitive information stored in the system.

#### 5.5.6. Secure Software Execution Environment

It refers to a protected, managed-code and runtime environment designed to protect against unexpected applications or software.

#### 5.5.7. Secure Contents

Content security makes information secure of the system and saves from attackers, viruses and intruders.

#### 5.5.8. Tamper Resistance

There are many attacks that affect the system and take information of users without their permission. Therefore, there is a need of such mechanisms to withdraw control from the attackers.

Due to all attacks and security requirements, an embedded security system is presented [103]. It captures information from the environment by using sensors. It also captures information that can affect the environment and send it to the master computer. The master computer performs precaution steps to save the information of users. The proposed architecture is also known as network based architecture, which has all features to fulfill the requirement of security.

There are many key features provided by the security framework and architecture. The details of features are as follows:

- Security: It provides security to the information of users in a form of lightweight cryptography. It is used to convert a message into cipher text to prevent attackers. It consumes less power and less memory to convert an original message into cipher text. It does not require high processing speed.
- Secure Operating System: It provides secure operations to ensure a secure communication between two parties by providing secure booting, secure execution environment and secure contents.
- Physical Protection: It provides physical security to the secret keys. The purpose of protecting it is to keep the secret keys from the attackers so that they cannot access the messages.
- Secure Storage: It protects the information of users stored in random access memory (RAM), read only memory (ROM) and any other secondary storage.

The features of the proposed framework include secure secondary storages, secure runtime execution environment and a secure memory management unit are the main focus for built-in security. Thus, it is a secure security architecture and framework for IoT.

### 5.6. Identity Management Framework

IoT demands security to perform its ability to work effectively. Identity management is one of them. Many devices are connected in the network and the network is also connected to other networks. The authentication is necessary for each device to communicate with each other. It provides help to check the authenticity of devices before sending or receiving the information.

There is a need for a mechanism to check whether the device is valid or not before sending or receiving information. To fulfill this need, a method is proposed named the identity management framework. It is comprised of four parts: environment, sensors, and receiver, and the foremost part is network. The environment refers to the place where sensors are attached to objects. The sensors have a responsibility to get information from objects. It sends all information to the master computers where the decision is taken. The receiver takes the result after performing the decision from the master computer. The last part is network, which is used to transfer the information. The information is transferred from sensors to master computers and also from master computers to receivers.

**Figure 13:**
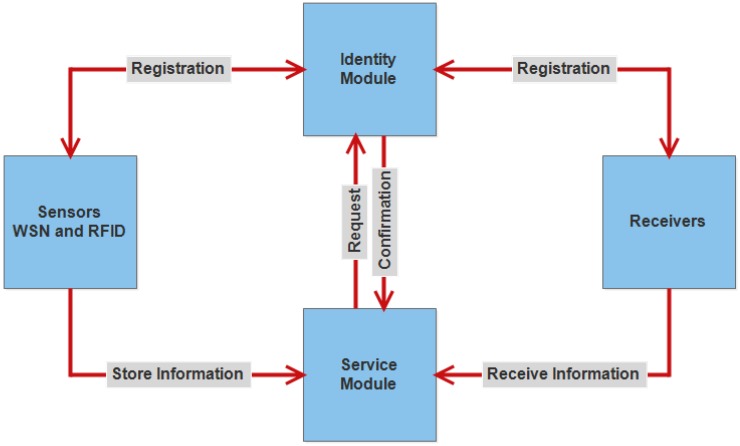
Identity management framework for cloud based IoT applications.

The proposed framework includes two segments: identity manager and service manager, as shown in Figure 13. The identity manager works to verify that the sensors and the receivers have rights to send and receive the information. The service manager provides services to devices after getting the authentication approval from the identity manager.

The functionality can be understood in this way that each sensor and receiver must be registered to use the framework. The identity manager assigns a unique key to them at the time of registration. It is necessary for each sensor to show a private key before sending the information. It is verified by the identity module to check whether the key is valid or not. It sends an approval message to a service module when the key is correct. After getting the message, the service module allows the sensor to save information. On the other side, it is also necessary for the receiver to show his assigned key to receive information. After verification by an identity module, it becomes able to receive the information from the service module [104].

### 5.7. Risk-Based Adaptive Framework

Due to security issues, the trust of users has been reduced. Another framework that can be helpful to reduce the number of security issues is a risk-based adaptive framework. It checks the environment after some intervals of time to find changes. If there is some change, it guesses the type of attack to decide whether it is a known or unknown attack. It also predicts the loss that can occur due to this attack. When attacks come, their type and the method used to avoid them are saved. A known attack refers to an attack that came at a previous time. The solution already exists to prevent these attacks. Therefore, these attacks cannot affect the system. Unknown attacks are new to the system. Their solution does not exist in the system. The master computer takes a precaution step to prevent these attacks immediately.

The fucntioning of the proposed framework is divided into four parts. The first part is adaptive risk management. It checks the environment constantly to find changes. If there is some change, it predicts loss that can occur due to this attack. It has some pre-defined instruction to protect the system from loss. The second part is adaptive security monitoring. It finds the type of attack. If the attack is known, it already has a solution to stop it. Therefore, attacks cannot affect the system. If the attack is unknown, it has no solution to prevent the attack. The third part is analytics and a predictive model. They handle the unknown attacks. They take precaution steps to prevent the attacks. They send all instructions to the fourth part whose name is adaptive security decision-making models. It makes a final decision and sends results to the device so that attacks could not affect the device [105].

### 5.8. Association of SDN with IoT

Due to heterogeneous characteristics, each device has different capabilities, software and hardware. Therefore, security has become a difficult task to implement on the IoT devices.

**Figure 14:**
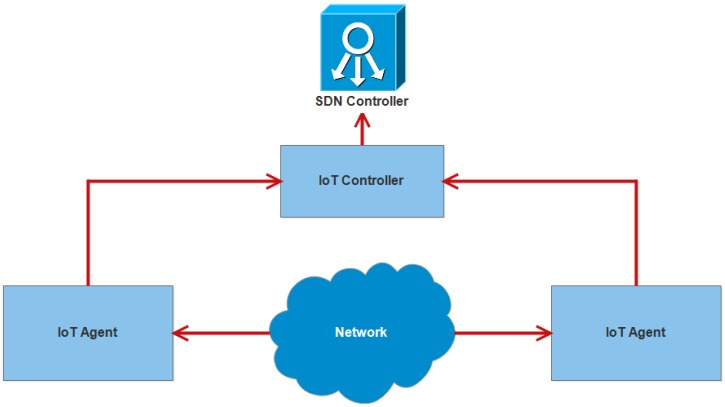
Association of SDN with IoT as a security solution.

To overcome these issues, many techniques have been proposed and the use of SDN [106] is one of them. It is used to eliminate the restrictions in traditional networks. It provides better performance at less cost and also lessens the cost of the network resources that are used in the network. Therefore, SDN is used as the association with the IoT to eradicate the issues regarding the security. Both technologies combine their architectures to make one architecture that has three devices: IoT agent, IoT controller and SDN controller. The architecture of SDN with IoT as a security solution is shown in Figure 14.

The IoT agent acts like a perception layer. It has a responsibility to check the environment constantly. If there is some change in environment, it collects information through different types of sensors. It also sends information to the IoT controller. Before sending the information, authentication is performed by both devices. The IoT agent checks authenticity before sending the information while the IoT controller also checks the authenticity before receiving the information from the IoT agent. There are many ways that can be used to authenticate the devices. The traditional way is the use of a pre-shared key or password. The other ways that can also be used are card scanning, voice and face recognition and fingerprint. There are several attackers that can receive and use the information of users according to their needs. Therefore, the authentication is very necessary before sending and receiving the information. The SDN controller works at the backend of this whole architecture. It manages and controls the security and provides the protection of all devices. If an IoT agent sends fake information to the IoT controller, it will stop the process and does not allow information to enter into the network. It provides security to a network layer so that fake information and the attacker could not enter. Therefore, the mechanism is implemented in the network layer [107].

### 5.9. Cooperation of Nodes Based Communication Protocol

The authors proposed a protocol based on ad hoc environment. The aim behind the proposed protocol is to detect the nodes that can affect the whole network through their misbehavior. There are four components maintained by each node, named the monitor, the reputation system, the path manager and the trust manager. The working of ad hoc based communication protocol can be understood in this way that each node monitors the behavior of all nodes that exist in a neighborhood. If a doubtful behavior is detected, the information of node along doubtful behavior is sent to a trust manager. The ALARM message is generated by a trust manager to inform all other nodes within its range. The ALARM message consists of information about the address of a reporter node, the address of an attacker node and packet loss. The node who receives an ALARM message evaluates whether the reporter node is authenticated or not as well as packet loss through a reputation system. If the provided information is correct, the path manager makes a new path from the source node to the destination node in order to provide reliability [108].

### 5.10. Reputation System Based Mechanism

To detect the misbehavior of a node in an ad hoc communication environment, the authors suggest a new protocol to detect an attacker node through collaboration of nodes. There are two entities maintained by each node: reputation table and the watchdog mechanism. The reputation table contains the information related to the receiving packets from other nodes. While the watchdog mechanism acts as a validation phase and is used to detect misbehavior or doubtful behavior of a node. The working of the proposed protocol can be understood in this way that whenever a node receives a packet from other nodes, it triggers a watchdog mechanism and stores the value in a temporary buffer and compares it with the observed result. If both are the same (calculated and observed), remove the calculated value from the buffer and come in the idle stage and wait for other observations as well. In case of no match, update the entry in the reputation table as a negative factor and inform all other nodes within its range about the behavior of a node [109].

### 5.11. Cluster Based Intrusion Detection and Prevention System

The authors proposed a cluster based intrusion detection and prevention system. In the proposed scheme, the network is divided into clusters and each cluster has a cluster head selected by a base station. The parameter used to select a cluster head is energy and trust level of a node. The parameters used to compute the trust level of nodes are packet generating rate, packet receiving rate and packet sending rate The proposed scheme is hierarchical and consists of two levels: cluster level intrusion detection (CLID) and network level intrusion detection (NLID). In CLID, the cluster head has a responsibility to compute the trust level of all nodes existing within its range. If a node has malicious behavior according to the trust level, it neglects that node and stops sending and receiving packets through it. The second level is NLID, where a cluster head sends all collected data to a base station in order to compute trust level. If the base station finds any malicious behavior, stop that specific node for further activities related to forwarding and sending packets in network [110].

### 5.12. Preference Based Privacy Protection Method

**Figure 15:**
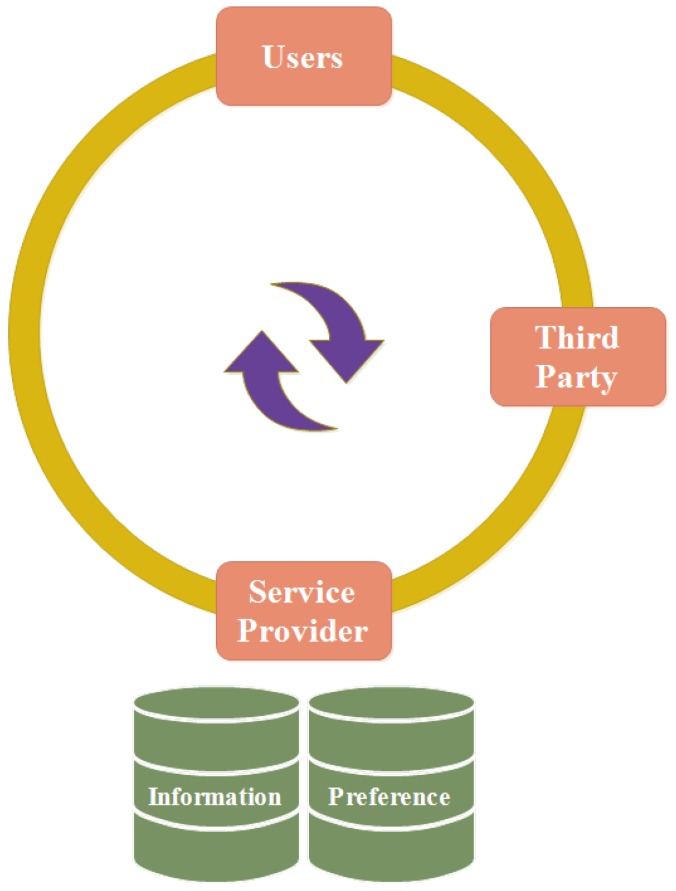
Preference based privacy protection system for IoT.

In the starting age of IoT, this method was used in small applications to get common information from users like name, age, gender, etc. Therefore, issues of security and privacy were not considered [111]. Due to development in IoT, it gets personal and private information of users from which issues of security and privacy were raised [112]. To keep the trust of users on IoT, security and privacy shall be considered. Therefore, a new mechanism is proposed that provides privacy protection based on preference as shown in Figure 15. The research is not complete, but it is a foundation for future research. In this context, preference means priority. A third party organization works as a bridge between the service provider and the users.

The working of the mechanism can be understood easily in the way that a service provider assigns an identification level to each piece of information that shows how much it should be secured. For example, a national identity card number demands a high identification level, which means it requires high security. It also tells how long the information is stored by it. It makes a table in which both identification level and its time are mentioned to send the users through a third party organization. The user checks all tables and sends the feedback to the service provider through third party organization. The service provider makes changes according to the user’s feedback.

There are two types of databases: privacy information and privacy preference. Privacy information is used to store the information of users like name, national identity card number, current location and cell phone number. Privacy preference is used to store the identification level of all information. The service provider manages security as well as privacy of user’s information. It has no rights to share the information of users to any other organization. It sends information after getting permission from the users. The third party organization acts like a supervisor and has the right to provide punishments or rewards according to the feedback that is given by the users. It also has the right to check how the information of users is stored to make it secure [113].

### 5.13. Access Control Mechanisms

At the time of designing the architecture of IoT, there exist some challenges that must be considered. The first issue is massive scaling. The network has a large number of devices. These devices need an identity to communicate with each other. Therefore, supports of a large amount of devices have become a challenge in IoT. The second issue is dependency because devices do not have their own architecture. Therefore, devices depend on other devices to communicate and connect [114].

In IoT, big data has also become an issue of generating a large amount of information with a lot of noise. Security is another issue that needs to be solved. There are many things that can cause security issues. Software errors can affect the security of IoT and come out due to the mistake of programmers. There are many attacks on the network that can affect the information of users like man-in-the-middle attack and denial of service (DOS) attack.

To overcome the issue of security, authentication and access control mechanisms are used. The process of verifying the user’s identity is called authentication. It demands credentials from users that are matched to those on file in a database of authorized user’s information on a local operator or within an authentication server. If the credentials match, the procedure is completed and the user is granted access. It entails the use of a username and password but other ways to authenticate can be through cards, retina scan, voice recognition and fingerprint. The access control mechanism is also used to reduce the issue of security. It is a security technique that is used to control and manage users as well as resources. There are two categories of access control: physical and logical. Physical access control limits access to buildings, shops, rooms and campuses while logical access control limits connections to networks, files and data [115].

### 5.14. OpenHab Technology

IoT provides many conveniences and benefits to humans, but, on the other side, it also has some drawbacks. These drawbacks make IoT unsafe. The most important drawback is security that users tolerate. Due to security, it has become untrustworthy for the users. Therefore, users are moving to another platform of IoT, which is known as OpenHab. It is an open source platform that is used for applications of IoT. It acts like a server and it must be installed on the computer to use. It is necessary for the devices to communicate that they first register for the proposed technology. The devices send a request for the registration. Before approving a request, it checks capabilities of the devices. It also checks the type of software and hardware. It registers the devices that meet its requirements. It sends a refuse message to the devices that do not have capabilities. Thus, it also removes the issue of device mismatch. It allows the devices that have the same capabilities, software and hardware. It provides security but it supports limited devices to prevent issues of device mismatch [116].

### 5.15. IoTOne Technology

OpenHab provides security, but, on the other side, there are many issues related to it, such as device incompatibility and it does not provide user friendly environments. To solve these issues, IoTOne is presented.

#### 5.15.1. Device Compatibility

It offers a heterogeneous solution to resolve the issue of device compatibility. It provides a facility to host IoT devices from different vendors. IoT vendors support an open-source environment and third party applications. It permits all IoT devices that have an ability to run the Internet. Therefore, it provides consumers with a larger selection of IoT devices to select which possibly results in lower deployment cost and more customizations.

#### 5.15.2. User Friendly Environment

Most people do not have time or knowledge to invest in learning how to use and set up an IoT system. Therefore, IoTOne provides a user friendly environment that allows consumers to begin work and communicating with their other devices. It also provides a simple, easy and user friendly way for process of device registration, downloading applications from its server and controlling the downloaded applications.

#### 5.15.3. Security

IoTOne provides the facility to host open-source and third party applications. Therefore, it should make sure that developers of third party applications do not use unsafe methodologies in their backend program to harm the system. Hence, third party developers must submit his backend program for his third party application to the IoTOne system for security testing [116].

### 5.16. Virtual Identity (VID) Framework

In IoT, the user’s information, such as personal home information, health information and hobbies, is collected, stored, processed and transmitted. If measures of information protection are not taken, the information of users may be revealed and accessed by unauthorized users.

The concept of virtual identity (VID) is put forward and a virtual identity framework is presented. It is used to solve privacy problems so that user’s information remains safe from unauthorized users. The user sends a request of VID to service provider. The service provider demands user’s information, such as name, age, gender, telephone, qualification, profession, driving license and a passport, in order to issue the VID for the user. The VID is provided to the user. The provider can issue a user more than one VID, which is not linked to another. It stores the information of a user along a VID. The information of users can only be tracked by the VID provider. The user accesses the network to use applications with VID. The provider of VID has a responsibility to control and manage the user’s information among different platforms. It does not provide information to anyone without permission of the user. VID is not accessible by attacker and it is an impossible task to access the information of user without VID. Hence, the VID framework ensures the privacy of user’s information and saves from attackers and unauthorized access [117].

### 5.17. Identity-Based Personal Location System

There is a need to make such a system that can be used to detect a user’s location in emergency cases through IoT. It demands high security because users commonly prefer their location information to be kept secret. Therefore, only authorized entities should have access to users’ location information and only when essential. For user’s privacy, the information of users about location should not be transported in clear text. It must be transferred in an encrypted form so that anyone would not be able to see the location.

**Figure 16:**
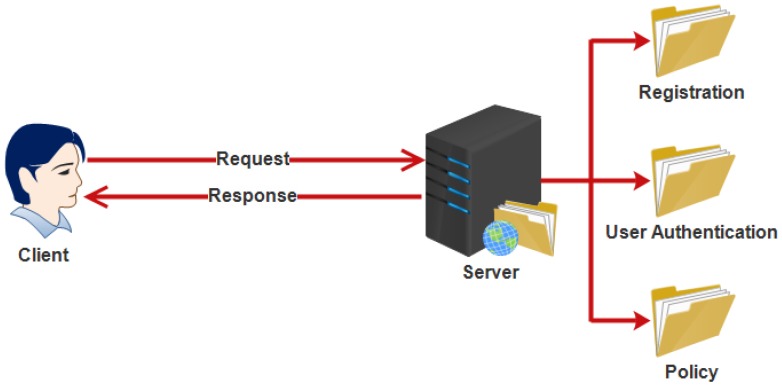
Identity based personal location system for IoT.

To fulfill the needs, a location system is proposed. The system includes the following four subsystems: registration subsystem, user authentication sub system, policy subsystem and client subsystem as shown in Figure 16.

#### 5.17.1. Registration Subsystem

The registration subsystem is used for registration of users. The user sends a request to the registration subsystem that includes the information of user, such as name, age, gender, password, mobile, qualification, profession, etc. It creates a VID for the user and saves the information of user. The client only keeps the VID of users, not the true information of the user.

#### 5.17.2. User Authentication Subsystem

A user authentication subsystem is used to verify the identity of user. In an emergency situation, a user sends a request to get the location of target user (who can help). Therefore, an identity must be checked before to give the location of target user. It receives the VID of the user and checks the identity.

#### 5.17.3. Policy Subsystem

Policy subsystem is used to provide the policies needed in the system to perform responsibilities properly. Admin can add new policies in the system and also delete existing policies from the system.

#### 5.17.4. Client Subsystem

The client system runs on the mobile device. It is used by the user to communicate and coordinate to the server. VID is also kept in the client subsystem [118].

Table 4 summarizes the existing methods and techniques regarding the security used in order to provide protection to the IoT applications.

## 6. Improved Layered Architecture of IoT

The architecture of IoT is different from architecture of Internet and Telecommunication Networks [119]. Therefore, the above stated three different architectures of IoT are not suitable for IoT applications, although they have some common features. They do not fulfill the requirements of security and privacy and are affected by numerous security attacks. To overcome the issue of security, we suggest and establish a new and generic layered architecture of IoT that has six layers. It can be easily and smoothly extended to basic functions with little impact on existing layered architectures of IoT to enforce the security attacks and prevent IoT applications from attackers. The names of layers of the new proposed architecture of IoT are:

- Perception Layer
- Observer Layer
- Processing Layer
- Security Layer
- Network Layer
- Application Layer

**Figure 17:**
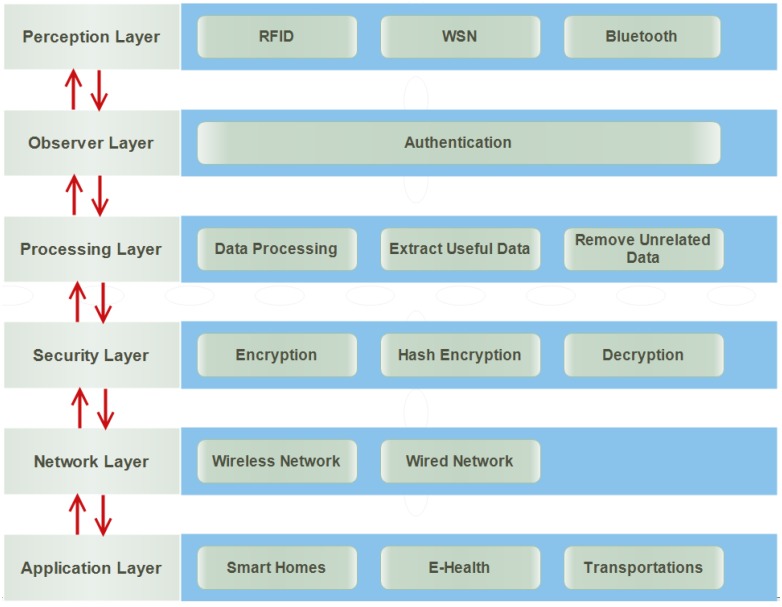
An improved layered architecture for IoT.

The responsibilities of these layers are as follows and Figure 17 shows the new improved layered architecture of IoT.

### 6.1. Perception Layer

It is also known as a sensor layer or physical layer. It acts like five organs of IoT. It identifies objects in order to gather information. For this purpose, different types of sensors are attached to the objects such Radio Frequency Identification (RFID) tags, barcode, Bluetooth, wireless sensors, LTE, etc. A sensor is chosen according to the needs of users and objects where it is attached. It sends collected information to the observer layer to check the authentication of these sensors and devices.

### 6.2. Observer Layer

The observer layer is also known as a monitor layer. The perception layer sends information to the observer layer. It checks information about whether it is protected from intruders and viruses or not. If there is any attack, it does not pass information to the next layer for further processing. It only passes that information that is protected from intruders and viruses. Furthermore, it also checks authentication of the objects. There are many ways to prove the identity such as authentication.

### 6.3. Processing Layer

It collects information from the observer layer. It trusts that the information provided by the observer layer is protected from every type of attack. This layer is designed to eliminate unnecessary information. It stores, analyzes and processes a huge amount of information that comes from the observer layer. It uses various technologies to extract useful information such as databases, cloud computing and data processing modules. The reason of removing unnecessary information is to save the network from heavy traffic. It also saves storage devices so that they cannot cross their limits.

### 6.4. Security Layer

There are many proposed architectures of IoT, but they do not have a layer regarding security. It is designed to make the architecture of IoT secure. There are many attacks on the network layer trying to get information from the users. Therefore, it makes secure information before sending to the network layer. It receives information from the processing layer. It performs encryption through converting all information collected from the processing layer into unknown form, called cipher text. The process of encryption is performed by using keys. It sends encrypted information to the network so that could not be understood by anyone other than the authentic users. It also sends a key to the receiver to convert the cipher text into original text. Thus, this layer protects the information of users from the attackers and risks existing on the network layer. There are many ways to encrypt and decrypt the information such as Advanced Encryption System (AES) and Data Encryption System (DES).

### 6.5. Network Layer

Network layer is also known as transmission layer. The role of it is to connect all things together and permit the sharing of information to other connected things. It receives information from the security layer in the form of cipher text. The reason for receiving information in cipher text is to protect from attackers and risks. The medium of transmission can be both wireless media and wired. The medium is selected according to the needs of users and also communication technologies.

### 6.6. Application Layer

Application layer is the last layer of the newly proposed layered architecture. It is responsible for data formatting and presentation. It is also responsible for delivery of numerous applications to different users. It defines several applications in which IoT can be used, such as smart home, smart transport, smart cities, smart health, animals and agriculture. It has a responsibility for providing the application specific service to the users. The service is chosen according to the information that is collected by the sensors from objects.

## 7. Key Challenges and Future Directions

**Figure 18:**
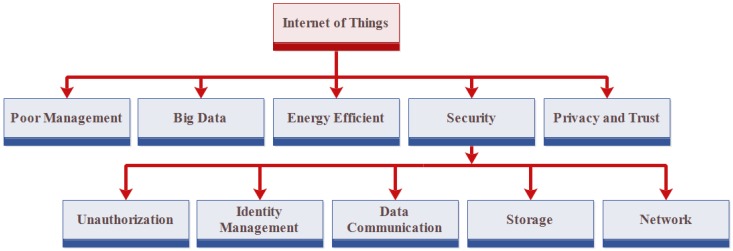
Research challenges and future directions in IoT.

The IoT offers enormous economic benefits, but it also faces many key challenges. The aim of this section is to provide the research directions for the new researcher in the domain. This section discusses the challenges remaining to be addressed for accommodating the trillion of IoT devices. Some of them are briefly discussed below and also shown in Figure 18.

### 7.1. Poor Management

Poor management has become a challenge for the IoT based applications. The issue is that developers focus on getting useful information from the objects by sensors. They do not pay attention to how information will be obtained. Due to uncertainty, attackers can access the information of users and use it according to their needs. Therefore, developers must change their aim and also focus to how they will get information.

### 7.2. Naming and Identity Management

Each device needs a unique identity to communicate in the network. Therefore, there is a need for the mechanism to assign a unique identity of each object dynamically in the network [120]. In the starting period of IoT, IPv4 was used to assign a unique identity in the network. Due to increasing the number of IoT based devices, IPv6 is used to assign the identity.

### 7.3. Trust Management and Policy

Trust is a very important and complex concept. It demands not only security but also many other things—for example, scalability, reliability, strength and availability. It has a bigger scope than security. The users provide their private information to the applications of IoT. Therefore, privacy must be provided. Privacy means the information of users is secure and cannot be accessible to others. Many techniques in research papers have been published by researchers to provide trust and privacy. These techniques have failed to provide trust and privacy to the applications of IoT. Therefore, these have become major challenges of IoT and must be solved in future research [121].

### 7.4. Big Data

Billions of devices are currently associated with the Internet forming the Internet of Things (IoT). These devices are generating an enormous amount of information. The transmission and processing of big data is a challenging task of IoT. Therefore, there is a need for such a mechanism that can solve the issue of big data.

### 7.5. Security

The security of information is a challenging task in IoT. The users send private information to fulfill their tasks. There are many attackers that can access the user’s private information. Therefore, there should be mechanisms to make the information of users secure so that attackers can not access it [122,123,124].

### 7.6. Storage

Secure storage has also become a challenge in IoT. The information is captured from objects by using sensors and is sent to storage devices. There is no encounter measurement to make storage devices secure. Therefore, there should be a mechanism to prevent the information from external monitoring or attackers.

### 7.7. Authentication and Authorization

There are many ways to authenticate the users. The traditional way is the use of a username and password, but other ways can be through access cards, retina scan, voice recognition and fingerprints. Authorization can also be achieved by defining the access control. It is a security technique that can be used to control and manage who or what can view or use resources of a system. Due to large number of objects in the network, it has become complex. Therefore, traditional ways of authentication and authorization have failed in the large network. Although research has attempted to solve the issues of authentication and authorization [125,126], some issues still exist. There is a need of such a mechanism by which these challenges can be solved [127].

### 7.8. Secure Network

There are many attacks in the network layer, for example, denial of service (DoS) and man-in-the-middle attack. A DoS attack is a security event that happens when an attacker takes action that prevents legitimate users from accessing targeted systems, devices or other network resources. A man-in-the-middle attack is a type of cyber-attack in which an attacker secretly interrupts and transmits messages between two parties who trust that they are communicating directly with each other. Therefore, there should be some mechanisms that provide security to a network layer [128].

## 8. Conclusions

The emerging idea of Internet of Things (IoT) is quickly finding its path throughout our modern life, aiming to enhance the quality of life by connecting various smart devices, technologies and applications. Generally, the IoT would allow for the automation of everything around us. This paper presented an overview of the premise of this concept and its applications. We have articulated different research about layered architectures of IoT and also described security attacks based on the layers that can affect the performance of IoT. The communication technologies have been presented with their features and limitations. We have surveyed the literature on the existing mechanisms to protect the IoT infrastructure and summarized these security methods on how they address the security issues in the IoT. We have also summarized the restrictions and limitations of the existing security methods. We also proposed a new layered architecture having six layers to make secure the infrastructure of IoT. Furthermore, several open research challenges associated with the IoT technology have been discussed as future directions. These challenges need to be addressed and implemented immediately.

## Figures and Tables

**Table 1:** Comparison of different application domains of IoT.

	Home/Office	City	Transportation	Agriculture	Retail
**Number of Users**	Very Few	Many	Many	Few	Few
**Communication**	RFID and WSN	RFID and WSN	WSN	WSN	RFID and WSN
**Network**	Small	Medium	Large	Medium	Small
**Internet**	Wi-Fi, 3G, 4G	Wi-Fi, 3G, 4G	Wi-Fi, Satellite	Wi-Fi, Satellite	Wi-Fi, 3G, 4G
**Bandwidth**	Small	Large	Medium	Medium	Small
**Test Beds**	Smart Home	Smart Cities	Few	PSCM System	Retail centers
	[13]	[14,15]	[16]	[17]	

**Table 2:** The elements and key technologies of IoT.

IoT Elements		Technologies
Identification	Naming	Electronic, Product Code, Ucode
	Addressing	IPv4, and IPv6
Sensing		Smart, Sensors, RFID Tags, Wearable Sensing Devices and Actuators
Communication		Radio Frequency Identification, Wireless Sensor Network, Near Field Communication (NFC), Bluetooth, Long Term Evolution (LTE)
Computation	Hardware	Audrino, Raspherry Pi, Intel Galil
	Software	Operating System
Services		Identity-Related, Information Aggregation, Collaborative-Aware and Ubiquitous
Semantics		RDF, OWL, EXI

**Table 3:** Comparison of different communication technologies used in IoT.

Technologies	Mechanism	Security	Applications	Characteristics	Drawbacks
ZigBee [57,58,59,60,61]	Wireless	Encryption, ntegrity	Home and Industry	Low consumption, Cheap	Fixed key
Bluetooth [62,63,64,65]	Wireless	Encryption, Authentication	PDA, Mobiles and Laptops	Cable replacement, Low cost	Blue jacking, Bluesnarfing
RFID [66,67,68,69,70]	Frequency waves	Encryption (AES, DES)	Health care	Data capturing with no duplication	No authorization
WSN [71,72,73,74,75]	Wireless	Key, Encryption, Authentication	Buildings and Health care	Low Cost, Power, and Resilience	DOS attack
Wi-Fi [76,77,78,79,80]	Radio Signals	Authentication, Authorization	PC, Phones and Cameras	Faster, Secure, Convenient	Eavesdropping
5G Network [81,82,83,84]	Wireless	Authentication, Authorization	Phone, IoT and Multimedia	Faster, Secure, Convenient	Distributed DoS

**Table 4:** Comparison of existing mechanisms along description with respect to security for IoT.

Method’s Name with Layer	Description	Issues Which It Address
Hashed Based Encryption [87] in Perception Layer	Hash Functions are used along encryption algorithms.	It is used to check the integrity of the message.
PKI protocol [89] in Perception Layer	Base station sends message to destination and has the public key.	It does not compromise about security so, deliver message by itself.
Secure Authorization Mechanism [90,91] in Perception Layer	Client - Server based System. It consists of two mechanisms; RBAC and ABAC.	Client send a request to server in order to fetch required resources. As a result, client get resources from server in a secure way.
Lightweight Cryptographic Algorithms [92] in Perception Layer	Keys are used to convert messages.	It is used to convert a message from plain text to cipher by using symmetric, asymmetric key and hash functions.
Embedded Security Framework [102,103] in Perception Layer	It provides not only security but also secure OS, memory and run time environment.	It provides secure secondary storage, run time environment and secure memory management in order to provide security to users.
Identity Management Framework [104] in Network Layer	It has two fragments of it; identity and service and Communicate via them.	It confirms from identity module which has information of users in order to prevent the attacker.
Risk based Adaptive Framework [105] in Network Layer	Four portions an each portion do their tasks and send the responsibility to other.	It stores the information about attack so when attacks come again, remove the attacks at second portion.
SDN with IoT [107] in Network Layer	SDN is used for better performance in low cost and use less hardware resource.	All communication is occurred by SDN which provides security to both; the IoT IoT agent and controller.
Cooperation of Nodes based Comm Protocol [108] in Network Layer	Node sends information to a trust manager to prevent the network from the intruders	It works on ad hoc communication environment. It detects and prevents the intruders.
Reputation System based Mechanism [109] in Network Layer	Node maintains two data structures; the reputation table and watchdog mechanism to detect intruders.	It works on ad hoc communication environment. It prevents the intruder the reputation system.
Cluster based Intrusion Detection and Prevention System [110] in Network Layer	Detects intruder by computing trust level. Trust level depends on packet generating, sending and receiving ratio.	It detects and prevents the intruder by dividing the network into cluster.
Preference Based Privacy Protection [113] in Application Layer	Communication occurs by service provider, client and third party in secure environment.	A third party organization acts like a bridge between service provider and client. It also checks security provided by the service provider to client.
Access Control Mechanism [115] in Application Layer	Simple Mechanism in order to provide security to users.	
OpenHab [116] in Application Layer	Provide security so people started to use it.	Simple registration but does not support device mismatch.
IoTOne [116] in Application Layer	Solve the issues occurred in the OpenHab Technology	Clients send the request to server in order to verify a user and provide the service by itself and also allow device mismatch.
Identity based Security Framework [117,118] in Application Layer	It consists of four subsystem; registration, user authentication, policy and client.	Policy based Framework that controls and manages users as well as resources. Polices are described by the Admin
